# Critical Roles of NF-κB Signaling Molecules in Bone Metabolism Revealed by Genetic Mutations in Osteopetrosis

**DOI:** 10.3390/ijms23147995

**Published:** 2022-07-20

**Authors:** Eijiro Jimi, Takenobu Katagiri

**Affiliations:** 1 Laboratory of Molecular and Cellular Biochemistry, Division of Oral Biological Sciences, Faculty of Dental Science, Kyushu University, 3-1-1 Maidashi, Higashi-ku, Fukuoka 812-8582, Japan; 2 Oral Health/Brain Health/Total Health Research Center, Faculty of Dental Science, Kyushu University, 3-1-1 Maidashi, Higashi-ku, Fukuoka 812-8582, Japan; 3 Research Center for Genomic Medicine, Division of Biomedical Sciences, Saitama Medical University, 1397-1 Yamane, Hidaka-shi, Saitama 350-1241, Japan; katagiri@saitama-med.ac.jp

**Keywords:** osteopetrosis, NF-κB, NEMO, RelA (p65)

## Abstract

The nuclear factor-κB (NF-κB) transcription factor family consists of five related proteins, RelA (p65), c-Rel, RelB, p50/p105 (NF-κB1), and p52/p100 (NF-κB2). These proteins are important not only for inflammation and the immune response but also for bone metabolism. Activation of NF-κB occurs via the classic and alternative pathways. Inflammatory cytokines, such as tumor necrosis factor (TNF)-α and interleukin (IL)-1β, activate the former, and cytokines involved in lymph node formation, such as receptor activator of NF-κB ligand (RANKL) and CD40L, activate the latter. p50 and p52 double-knockout mice revealed severe osteopetrosis due to the total lack of osteoclasts, which are specialized cells for bone resorption. This finding suggests that the activation of NF-κB is required for osteoclast differentiation. The NF-κB signaling pathway is controlled by various regulators, including NF-κB essential modulator (NEMO), which is encoded by the *IKBKG* gene. In recent years, mutant forms of the *IKBKG* gene have been reported as causative genes of osteopetrosis, lymphedema, hypohidrotic ectodermal dysplasia, and immunodeficiency (OL-EDA-ID). In addition, a mutation in the *RELA* gene, encoding RelA, has been reported for the first time in newborns with high neonatal bone mass. Osteopetrosis is characterized by a diffuse increase in bone mass, ranging from a lethal form observed in newborns to an asymptomatic form that appears in adulthood. This review describes the genetic mutations in NF-κB signaling molecules that have been identified in patients with osteopetrosis.

## 1. Introduction

Bone is a dynamic tissue that is constantly being remodeled to maintain a healthy skeleton. This is essential for the efficient and lifelong performance of important skeletal functions [1,2]. Bone remodeling is a physiological process in which old or damaged bone is resorbed by osteoclasts and then replaced by new bone formed by osteoblasts [3,4]. In normal bone remodeling, the balance between bone resorption and bone formation is tightly controlled by hormones and local factors, such as cytokines and growth factors, to maintain bone mass and mechanical strength. Nevertheless, an imbalance between bone resorption and bone formation can occur under certain pathological conditions [3,4]. For example, in osteoporosis and rheumatoid arthritis, bone resorption exceeds bone formation, resulting in a decrease in bone mass. In contrast, osteopetrosis is a pathological condition in bone that is characterized by a diffuse increase in bone mass caused by a reduction in bone resorption.

**Figure 1:**
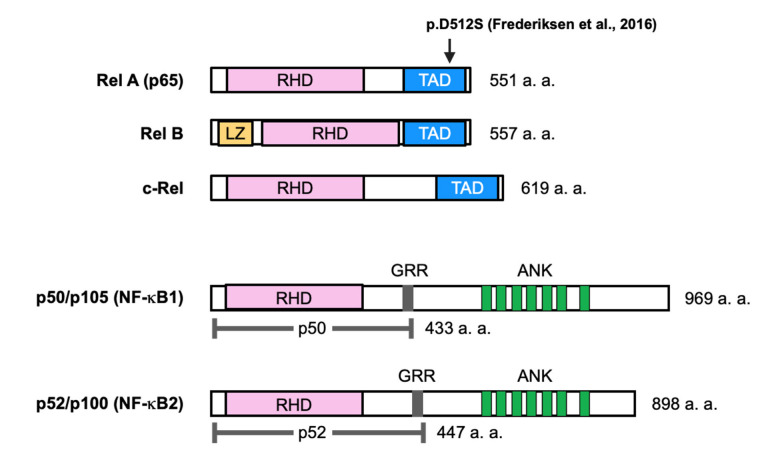
Schematic representation of the NF-κB family proteins. Members of the NF-κB protein family are shown. The total number of amino acids in each protein is indicated on the right. Presumed sites of cleavage in p105 (amino acid 433) and p100 (amino acid 447) are indicated by dotted lines. RHD: Rel homology domain; TAD: transcriptional activation domain [8]; LZ: leucine zipper; GRR: glycine-rich repeat; ANK: ankyrin repeat. An arrow indicates the position of a genetic mutation associated with osteopetrosis.

**Figure 2:**
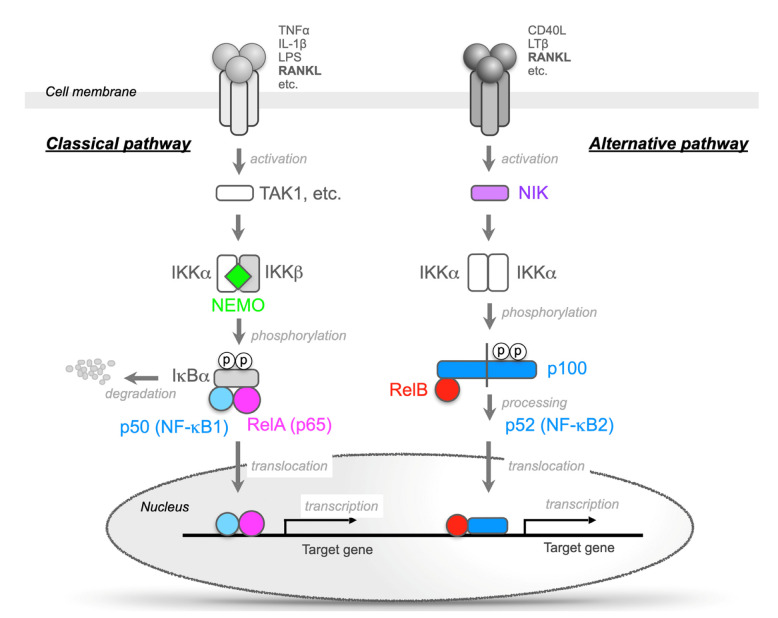
Two different NF-κB signaling pathways. The classic (canonical) pathway (**left**) is activated by a large number of agonists, such as TNF-α, IL-1, lipopolysaccharide, and T-cell receptors. The activation of this pathway depends on the inhibitor of the κB (IκB) kinase (IKK) complex (IKKα/β and NEMO), which phosphorylates IκBα (Ser32, 36) to induce rapid degradation. This pathway is essential for immune responses, inflammation, tumorigenesis, and cell survival. The alternative (noncanonical) pathway (**right**) is activated by a limited number of agonists, which are involved in secondary lymphoid organogenesis. This pathway requires NF-κB-inducing kinase (NIK) and IKKα. These kinases induce the slow processing of p100 to generate p52, resulting in the dimerization and activation of the p52/RelB heterodimer. The activation of NF-κB signaling stimulates osteoclastic bone resorption and suppresses bone formation.

Nuclear factor-κB (NF-κB) was identified as a transcription factor that binds to the enhancer of the κ light chain of immunoglobulins and is selectively expressed in B cells [5]. The NF-κB family of transcription factors consists of five ubiquitously expressed proteins in mammals, RelA (p65), c-Rel, RelB, p50/p105 (NF-κB1), and p100/p52 (NF-κB2), which form various homodimers and heterodimers (Figure 1). The NF-κB signaling pathway is activated by ligands, such as tumor necrosis factor-α (TNF-α), interleukin-1β (IL-1β), lipopolysaccharide (LPS), and receptor activator of NF-κB ligand (RANKL), through transmembrane receptors. These receptors induce downstream signaling through inhibitor of κB (IκB) kinases (IKKs) a and b and NF-κB essential modulator (NEMO) (also known as IKKγ) (Figure 2) [6,7].

NF-κB is not only involved in inflammatory and immune responses but also plays an important role in the regulation of bone metabolism [6,7]. Genetic mutations in molecules involved in the NF-κB signaling pathway in mammals cause pathological bone phenotypes, including osteopetrosis. This review describes the known genetic mutations in NF-κB signaling molecules and neonatal osteopetrosis.

## 2. Regulatory Mechanism of NF-κB Signaling

The five members of the NF-κB family share a 300 amino acid N-terminal domain known as the Rel homology domain (RHD). The RHD is derived from the retroviral oncoprotein v-Rel, is involved in DNA binding and dimerization, and is associated with IκB proteins (Figure 1). Three members of this group, RelA, c-Rel, and RelB, contain C-terminal transcriptional activation domains (TADs). TADs are important because of their ability to induce target gene expression. The activation of NF-κB signaling is regulated by IκB proteins (e.g., IκBα, IκBβ, and IκBε). The p52 and p50 forms are processed from the precursors p100 and p105, respectively. These precursors are characterized by multiple ankyrin repeat domains and the ability to bind NF-κB dimers [6,7].

In unstimulated cells, the five related proteins of the NF-κB family form dimers but remain inactive in the cytosol by forming complexes with IκB proteins [6,7]. The binding of ligands to receptors on the cell membrane stimulates the phosphorylation of the NF-κB and IκB complexes by the IKK complex, and the NF-κB dimers (mainly the RelA/p50 heterodimer) are then activated by the degradation of IκB (mainly IκBα) (Figure 2). This is called “the classic NF-κB pathway” [6,7]. The IKK complex consists of three subunits, IKKα (also known as IKK1), IKKβ (also known as IKK2), and NEMO (Figure 2). Both the IKKα and β subunits are catalytically active, and NEMO serves as a regulatory subunit. IKKβ is the dominant kinase involved in IκB phosphorylation, and IKKβ-deficient mice present a phenotype similar to that of RelA-deficient mice. These mice die at embryonic day (E) 13.5 because of severe liver damage due to massive apoptosis [9,10,11,12]. However, IKKα-deficient mice can survive for a month after birth but suffer from striking morphological defects, such as markedly hyperplasic epidermis. The observation that NEMO-deficient mice die at E12.5-E13.0 because of severe liver damage due to massive apoptosis suggests that NEMO is indispensable for the activation of NF-κB signaling [13,14].

Furthermore, there is also an NF-κB activation mechanism that is independent of IκB degradation. In the unstimulated state, p100 remains in the cytoplasm by associating with RelB. RANKL, CD40 ligand, and lymphotoxin b bind to their receptors and activate NF-κB-inducing kinase (NIK). The activation of NIK results in the activation of the IKKa homodimer, and the C-terminal end of p100 has the same function as degraded IκB. Then, a heterodimer of RelB/p52 is formed and translocates into the nucleus. This activation pathway is referred to as “the alternative NF-κB pathway” (Figure 2) [6,7]. RANKL activates both the classic and alternative pathways [6,7].

## 3. NF-κB Signaling Regulates Bone Metabolism

### 3.1. NF-κB Functions in Physiological Bone Resorption

NF-κB is deeply involved in various biological phenomena and is an important molecule in the regulation of bone metabolism [6,7]. Mice with double knockout of the p50 and p52 subunits of the NF-κB family proteins develop severe osteopetrosis due to a total lack of osteoclasts. This finding suggests that NF-κB signaling is indispensable for osteoclastogenesis [6,15,16]. Indeed, the osteoclast differentiation factor RANKL stimulates the activation of NF-κB in osteoclasts [17,18]. Mice deficient in either RANKL or its receptor, RANK, also exhibit severe osteopetrosis [19,20], and this phenotype is similar to that of the p50/p52 double-knockout mice. Furthermore, the loss of function of either RANKL or RANK in humans causes rare human osteopetrosis due to the loss of NF-κB signaling [21,22]. These findings suggest that the RANKL-RANK-NF-κB pathway is indispensable for osteoclast differentiation.

Among the NF-κB signaling molecules mentioned above, deficiency of p65, IKKb, and NEMO were lethal in early embryonic development in mice and could not be analyzed for bone phenotype [9,10,11,12,13,14]. Therefore, Ruocco et al. generated conditional knockout mice that were specifically deficient in IKKb in bone marrow cells (IKKbcKO) using Mx1- or CD11b-Cre transgenic mice [23]. IKKbcKO mice showed an increase in cancellous bone mass due to a reduced number of osteoclasts. Furthermore, the number of osteoclast progenitor cells (F4/80-positive cells) was significantly reduced. When IKKbcKO mice were crossed with TNFR1-deficient (TNFR1-/-) mice to generate IKKbcKO/TNFR1KOdKO mice, osteoclast progenitor cells were resistant to apoptosis. However, in IKKα knock-in (IKKαA/A) mice in which the serine residue required for IKKα kinase activity was replaced with alanine, osteoclast formation by RANKL stimulation was suppressed in vitro but not in vivo. The cancellous bone mass of IKKαA/A mice was similar to that of wild-type mice [23]. Therefore, IKKb, not IKKα, is important as a RANK downstream signal in osteoclast differentiation.

Since p65-deficient (p65-/-) mice are embryonic lethal [9], the number of osteoclasts was decreased in chimeric mice transplanted with p65-/- fetal hepatocytes into irradiated wild-type mice. When p65-/- chimeric mice were bred with TNFR1-/- mice, the p65-/- precursor was found to be sensitive to RANKL-induced apoptosis even in the TNFR1-/- background. A caspase inhibitor, ZVAD, restores RANKL-induced osteoclast formation in p65-/- precursors in vitro, indicating that p65 induces the expression of apoptosis-promoting genes in osteoclastogenesis [24].

It is well known that the alternative NF-κB pathway also involves RANKL-induced osteoclastogenesis. Administration of RANKL to NIK-deficient (NIK-/-) mice suppresses osteoclast formation compared to the administration of RANKL to wild-type mice. RANKL did not induce the processing of p100 to p52 in osteoclast progenitors derived from NIK-/- mice because p100 acts like IκB to prevent the formation of a RelB/p52 heterodimer [25]. Similar to NIK-/- mice, alymphoplasia (*aly/aly*) mice also do not undergo p100 to p52 processing. This is because NIK is inactive due to the loss of function caused by a point mutation [26]. *Aly/aly* mice showed mild osteopetrosis with a significant reduction in osteoclast number [27,28]. RANKL-induced osteoclast formation from bone marrow cells of *aly/aly* mice was also suppressed. RANKL still induced IκBα degradation and activated classic NF-κB, but processing from p100 to p52 was abolished by the *aly/aly* mutation. The overexpression of NFATc1 and the constitutive activation of IKKα or p52 restored RANKL-induced osteoclastogenesis in *aly/aly* cells. The overexpression of RelB in *aly/aly* cells restored RANKL-induced osteoclast formation by inducing Cot expression, which induces processing from p100 to p52 instead of NIK [29]. In summary, the balance between p52 and p100 determines RANKL-induced osteoclast formation.

### 3.2. Molecular Mechanisms of Osteoclast Differentiation Regulated by NF-kB

When RANKL binds to RANK, TNF receptor-associated factor (TRAF) family proteins are recruited to RANK, and among the TRAFs, the activation of NF-kB by TRAF6 is important for the induction of various genes [30]. The results of a DNA microarray indicated that the nuclear factor of activated T cells, cytoplasmic 1 protein (NFATc1), was one of the target genes of RANKL [31]. NFATc1 together with c-Fos induces the expression of osteoclast-specific genes, such as TRAP and calcitonin receptors. Induction of NFATc1 has been shown to be impaired in TRAF6-/- cells [31], suggesting that NFATc1 is one of the key target genes for NF-κB in the early stages of osteoclast formation. A specific inhibitor of NF-κB suppressed RANKL-induced osteoclastogenesis by suppressing NFATc1 expression [32]. Furthermore, there is a κB binding site in the NFATc1 promoter. NF-κB is recruited to the NFATc1 promoter shortly after RANKL stimulation, and the overexpression of NF-κB promotes NFATc1 luciferase activity [33]. Taken together, these findings indicate that it is important for NF-κB to induce NFATc1 immediately after RANKL stimulation.

### 3.3. NF-κB Negatively Regulates Bone Formation

NF-κB regulates osteoblastogenesis as well as osteoclastogenesis [6,34,35,36,37]. The inhibition of the classic NF-κB pathway through the expression of a dominant negative form of IKKβ or NEMO specifically in osteoblasts leads to enhanced bone formation, while the constitutive activation of IKKβ impairs bone formation [34,35,36]. Inhibition of the alternative NF-κB pathway also promotes bone formation. RelB-deficient mice show a transient increase in bone formation [37]. RelB-deficient bone marrow stromal cells proliferated faster in vitro and enhanced osteoblast differentiation associated with increased expression of Runt-related transcription factor 2 (Runx2). RelB directly bound to the promoter in the *Runx2* gene and suppressed its promoter activity [37]. In addition to suppressing osteoclast formation, *aly/aly* mice show the renewal of bone formation with increased bone formation rate, mineral deposition rate, and osteoblast number by enhancing bone morphogenetic protein (BMP) signaling [38]. These results suggest that both the classic and alternative NF-κB pathways negatively regulate bone formation.

Bone marrow mesenchymal stem cells (MSCs) are pluripotent cells that can differentiate into various cells, such as osteoblasts, chondrocytes, and adipocytes [39]. Increased inflammatory mediators have been reported to inhibit osteoblast differentiation and bone formation in bone defects or damaged bone tissue that requires bone regeneration. The inflammatory cytokines TNFα and IL-17 activate IKKβ and activate NF-κB to suppress the osteoblast differentiation of MSCs [40]. However, the inhibition of NF-kB by IKKβ knockout cells in which the adenovirus vector Cre was introduced into the MSCs of IKKβ^flox^/^flox^ mice or by IKKβ specific inhibitors promotes the osteoblast differentiation of MSCs and bone formation. The activation of NF-κB suppressed osteoblast differentiation from MSCs by promoting the ubiquitination and degradation of β-catenin via the induction of Smurf1 and Smurf2, which is mediated by the IKKβ-NF-κB pathway. In addition, IKKβ serves as a β-catenin kinase that phosphorylates the conserved degron motif of β-catenin and primes the protein for β-TrCP-mediated ubiquitination and degradation. Therefore, adipogenesis is increased and bone formation from MSCs is suppressed [41]. Conditional knockout mice that were specifically deficient in IKKβ in MSCs using Prrx1-Cre transgenic mice showed increased bone mass in adult mice and reduced adipocyte formation. In humans, IKKβ expression in adipose tissue was also positively correlated with increased steatosis and increased β-catenin phosphorylation. These findings suggest that IKKβ is an important molecular switch that regulates the fate of MSCs.

### 3.4. Genetic Mutations of the NF-κB Signaling Molecule in Osteopetrosis

Although several lines of evidence have shown that the NF-κB pathway controls the function of both osteoclasts and osteoblasts in mouse genetic models, there are no reports of human systematic bone diseases due to mutations in the genes encoding NF-κB family members. Mutations in the *IKBKG* gene, which encodes NEMO, have been reported in X-linked anhidrotic ectodermal dysplasia with immunodeficiency with osteopetrosis (OL-EDA-ID). Some NEMO mutations have been reported. However, they have all been associated with reduced NF-κB activity, and the cause of osteopetrosis is thought to be the inhibition of bone resorption by osteoclasts [42,43]. RelA mutations have recently been reported as a possible cause of genetic disorders characterized by high bone mass for the first time [8].

## 4. Osteopetrosis

Osteopetrosis was first described by Alberts Schonberg in 1904. It is a pathological bone condition characterized by a diffuse increase in bone mass, ranging from a lethal form observed in newborns to an asymptomatic form that appears in adulthood [44]. Osteopetrosis clinically manifests with varying degrees of severity. The neonatal/infant type is characterized by severe bone marrow failure, cranial nerve symptoms, hydrocephalus, hypocalcemia, failure to thrive, and other manifestations from an early stage. Due to pancytopenia, infection and bleeding are likely to occur. Thus, the mortality rate is high until early childhood. The intermediate type develops in childhood and is characterized by various symptoms, such as fracture, osteomyelitis, deafness, short stature, and abnormal teeth, but bone marrow failure is not serious. Bone marrow failure is not observed in the late-onset type, which is often diagnosed as pathological fracture, osteomyelitis of the mandibular jaw, facial nerve paralysis, and other conditions. This type may be identified by chance in X-ray films obtained for other reasons [39,40,41]. The main cause of osteopetrosis is deficient osteoclastic bone resorption, which impairs osteoclast differentiation or osteoclast function. *TCIRG1, CLCN7, OSTM1, TNFSF11, TNFRSF11, PLEKHM1, CA2, LRP5, NEMO, KIND3*, and *CalDAG-GEF1* have been reported to be related to multiple genetic abnormalities related to osteoclast differentiation and function [45,46].

## 5. Structure and Function of NEMO and NEMO-Related Diseases

**Figure 3:**
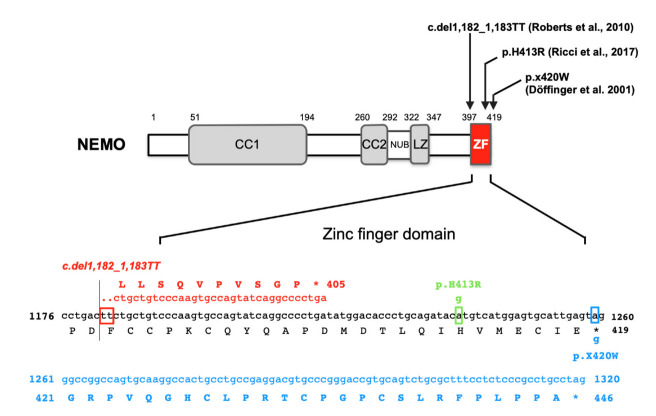
Schematic structure of NEMO and the genetic mutations in the *IKBKG* gene associated with osteopetrosis. The nucleotide and amino acid sequences of the *IKBKG* gene and the NEMO protein, respectively, are indicated in black (normal), blue (c.1,259A > G; p.X420W) [50], red (c.1,182_1,183del_insTT; OL-EDA-ID) [51], and green (c.1,238A > G; p.H413R) [52]. Dots indicate deletions, and open boxes indicate “stop codons”. CC: coiled-coil domain; NUB: NEMO ubiquitin binding; LZ: leucine zipper; ZF: zinc finger.

NEMO is an α-helix protein with a molecular weight of 50 kD that mainly contains two coiled-coil domains (CC), leucine zipper (LZ), and C-terminal zinc finger (ZF) regions (Figure 3) [6,7,42,43]. NEMO interacts with the IKK subunit through the N-terminal portion of CC1. It plays a central role in polyubiquitin-mediated IKK activation. This is because it specifically recognizes polyubiquitin sequences via the CC2-LZ region domain and is itself ubiquitinated. The gene encoding NEMO is located on Xq28 of the X chromosome and is not observed in other genes that encode known molecules in the NF-κB pathway. Diseases resulting from the development of mutations in *IKBKG* that affect X-linked genetic diseases due to the unique chromosomal position of this gene have been reported. *IKBKG* mutations are related to two clinically distinct hereditary genetic diseases, incontinentia pigmenti (IP, OMIM #308300) and anhidrotic ectodermal dysplasia with immunodeficiency (EDA-ID, OMIM #300291), depending on genetic status and their inhibitory effects on NF-κB activation. Genetic mutations that cause a complete lack of NEMO function are fatal in the embryonic stage in hemizygous males, but heterozygous females with IP disease survive due to X-chromosome inactivation. However, patients with EDA-ID are always male and hemizygous for *IKBKG* mutations that maintain persistently reduced NF-κB activation. EDA-ID is associated with susceptibility to life-threatening infection and ectodermal dysplasia, which is characterized by rare conical teeth, sparse scalp hair, frontal ridges, and a lack of sweat glands [42,43,47,48,49].

### 5.1. Incontinentia Pigmenti: IP

IP is a severe and extremely rare X-linked genodermatosis with an incidence of 1/10,000 to 1/20,000. It mostly presents in females, as males die during the fetal period. IP manifests in the skin, hair, teeth, nails, eyes, and central nervous system. It is caused by an *IKBKG* gene deletion from exon 4 to exon 10, potentially resulting in the production of a shortened ~130 amino acid protein. This truncated protein is unable to activate NF-κB, as observed in the approximately 60–80% of affected female patients who carry a deletion of 11 kb [49]. Many other small mutations (missense, frameshift, nonsense, and splice site mutations) have been reported [49]. A recent study revealed no significant differences in the three-dimensional analysis or biochemical parameters of the bones of female carriers with *IKBKG* mutations showing IP symptoms versus healthy controls [53].

### 5.2. EDA-ID

Patients with EDA-ID show ectodermal dysplasia and susceptibility to infection. EDA-ID shows X-linked recessive (XL-EDA-ID) and autosomal dominant (Ad-EDA-ID) modes of inheritance, and *IKBKG* and *IKBA*, which encode NEMO and IκBα, respectively, have been identified as causative genes [42,43].

### 5.3. Osteopetrosis, Lymphedema, Hypohidrotic ectodermal Dysplasia and Immunodeficiency (OL-HED-ID)

Figure 3 shows the *IKBKG* gene mutations associated with OL-HED-ID that have been reported thus far.

#### 5.3.1. p.X420W

Two unrelated male patients with a novel OL-EDA-ID syndrome who were the sons of mothers with mild IP died of overwhelming infections at 2.5 and 1.5 years of age, showing osteopetrosis, lymphedema, and EDA [50]. Sequencing analysis of peripheral blood genomic DNA from both patients revealed the same c.1,259G > A mutation in the *IKBKG* gene. The position of this mutation is the second nucleotide in the TGA stop codon. Therefore, this mutation destroys the stop codon, which instead encodes tryptophan at position 420 (p.X420W) (Figure 3). Moreover, the mutated allele adds 27 unique amino acids to the carboxyl terminus after the appearance of tryptophan in NEMO (Figure 3). The transcriptional activity of the wild-type and the mutant p.X420W has been examined in NEMO-deficient mouse cell lines. The transcriptional activity of p.X420W induced by LPS was reduced to approximately half of that of wild-type NEMO. Although the expression of IKKα/β in immortalized patient-derived fibroblasts and B cells was found to be comparable to that in healthy controls, the expression of NEMO was barely detectable in the patient-derived cells. In addition, the stimulation of patient-derived B cells with TNFα and cycloheximide did not induce cell death. Thus, unlike the previously reported lethal intrauterine loss-of-function *IKBKG* mutation, the p.X420W mutation in the *IKBKG* gene suppresses NF-κB activity, but not completely. Furthermore, in two patients with the X420W mutation in the *IKBKG* gene showing mild osteopetrosis with substantial extramedullary hematopoiesis, osteoclasts were observed to remain in bone tissue sections. This finding indicates that RANK signaling was partially impaired or that other signaling pathways were activated by this mutation [50].

#### 5.3.2. c.1,182_1,183TT Deletion

A 6-year-old male with two thymidine deletions at nucleotides 1,182 and 1,183 of the *IKBKG* gene showing symptoms of OL-EDA-ID was reported [51]. Although a functional analysis of the mutation was not performed, it was hypothesized that a two-thymine deletion induced a frameshift and a truncation of the transcribed protein. The mutated allele encoded a 405 amino acid protein with 10 unique amino acids at the carboxyl terminus and a deletion of 25 amino acids, including the zinc finger domain (Figure 3). These findings suggest a significant impairment of NEMO function.

#### 5.3.3. c.1,238A > G (p.H413R)

The examination of a 3-month-old infant with persistent diarrhea and failure to thrive revealed no sweat glands in a skin biopsy and osteopetrosis, suggesting OL-EDA-ID [52]. Therefore, a genetic test for *IKBKG* was performed. A novel missense mutation, c.1,238A > G, was identified in exon 10 of the ZF domain of the *IKBKG* gene [46]. When the patient’s blood cells were stimulated with TNFα, IL-1, or LPS, the production of IL-6, which is one of the targets of NF-κB, was found to be significantly reduced based on ELISA results. The c.1,238A > G mutation causes the replacement of histidine with arginine at position 413 (p.H413R) in the ZF domain of NEMO (Figure 3). These findings indicate that the stability of the ZF folds may be impaired and that the protein recognition ability may be altered.

Since these are rare case reports of NEMO mutations, no detailed analyses of the bone tissue of osteopetrosis patients or studies using osteoclasts and osteoblasts have been performed. It remains unclear how each mutation in the *IKBKG* gene affects bone metabolism, especially in osteoclasts. However, osteoclast differentiation or osteoclast function is presumed to be suppressed in patients with OL-HED-ID and is presumed to be caused by *IKBKG* mutations for the following reasons: (i) NEMO is important for the RANKL-stimulated activation of NF-κB [54], (ii) osteoclasts are not formed in p50/p52 double-knockout mice [15,16], and (iii) myeloid cell-specific NEMO-deficient mice develop increased bone mass because of the inhibition of osteoclast formation [54].

## 6. Neonatal High Bone Mass (HBM) Caused by RelA Mutation

RelA is the best-studied NF-κB family protein and is considered to be the most important molecule in the mechanism of NF-κB activation [6,7]. RelA-deficient mice die due to massive apoptosis in the liver on E13.5 [9]. In addition to the activation mechanism of NF-κB shown in Figure 2, it has been reported that the methylation/acetylation and phosphorylation of RelA are important for the function of RelA. Furthermore, mutations in the RelA gene have been reported in many cancers, but there have been few reports of systemic diseases due to mutations in RelA.

Recently, a genetic mutation in RelA was found after sudden death in a neonate with HBM, according to radiography and skeletal histopathology, without abnormalities in the liver [8]. Initially, eight genes thought to potentially underlie osteopetrosis were examined in this patient, but no mutations were found. Finally, whole-trio-based exon sequencing demonstrated that the patient had a de novo heterozygous missense mutation (c.1,534_1,535del_insAG, p.D512S) in exon 11 of *RELA*, which encodes RelA (Figure 1). In addition, bone taken from the metaphysis of the distal femur was osteosclerotic, and there was no clear distinction between the cortex and trabecula. Picrosirius red staining showed a mixture of layered and woven bone throughout the specimen, but bone resorption by osteoclasts was normal. These findings suggest increased and abnormal bone formation in the patient. Stimulation of patient-derived fibroblasts with TNFα reduced the transcriptional activity of NF-κB relative to that in healthy control-derived fibroblasts [8].

The patient’s RELA variant resulted in the replacement of a negatively charged aspartic acid at position 512 with a polar but uncharged serine in RelA. This alteration could lead to a significant change in the three-dimensional structure of the protein and cause the loss of RelA function. The suppression of RelA function has been reported to increase bone formation. However, the molecular mechanisms of the enhancement of bone formation have not been sufficiently proven since only the histological analysis of bone has been performed. The p.D512S mutation in RelA appears to be involved in HBM disorder. However, further studies are needed to investigate how this RelA mutation regulates NF-κB signaling as well as the differentiation and function of osteoblasts and osteoclasts.

## 7. Conclusions and Perspectives

In this review, we described osteopetrosis-associated genetic mutations in two NF-κB family molecules, NEMO and RelA, which are encoded by the *IKBKG* and *RELA* genes, respectively. NF-κB signaling has been shown to be important for the regulation of bone metabolism based on in vitro experiments using cultured cells and in vivo experiments using mice [6,15,16,23,24,25,26]. However, no genetic mutations of the NF-κB family molecules have been reported in systemic bone diseases thus far. Among the many mutations reported in the *IKBKG* gene, only a few are associated with osteopetrosis. The ZF region of NEMO contains genetic mutations associated not only with osteopetrosis but also with other diseases. This finding suggests that the observed phenotypic variations are not determined simply by the strength of the NF-κB signal [42,43,48,49,50,51,52,53]. In recent years, a sequence recognized as an intron splice donor site between the fourth and fifth exons of the IKBKG gene in patients with EDA-ID has emerged to generate pseudoexons. In these cases, abnormal mRNAs, including pseudoexons, are produced in addition to the standard mRNAs produced in normal conditions. Then, serine-arginine-rich splicing factor 6 (SRSF6) binds to pseudoexons and induces exon inclusion containing pseudoexons. When SRSF6 is knocked down or inhibited with a CDC-like kinase (CLK) inhibitor known to activate SRSF family molecules by a phosphorylation reaction, wild-type NEMO is expected to be expressed. Then, the cytokine response ability will be restored, and this could also be effective for the treatment of symptoms of EDA-ID [55].

Osteopetrosis-associated genetic mutations in NF-κB family molecules are rare. Further studies using cells and animal models that mimic variants of the *IKBKG* or *RELA* genes will reveal the physiological functions of NF-κB signaling molecules in bone metabolism that have not yet been elucidated. Furthermore, as mentioned above, these mutations are expected to be applied to treatment approaches by clarifying the mechanism and function of such mutations.

## Data Availability

All data are contained within the manuscript.
